# The Efficacy of Molecular Markers Analysis with Integration of Sensory Methods in Detection of Aroma in Rice

**DOI:** 10.1155/2013/569268

**Published:** 2013-10-09

**Authors:** H. Y. Yeap, G. Faruq, H. P. Zakaria, J. A. Harikrishna

**Affiliations:** ^1^Institute of Biological Sciences, Faculty of Science, University of Malaya, 50603 Kuala Lumpur, Malaysia; ^2^Centre for Research in Biotechnology for Agriculture, University of Malaya, 50603 Kuala Lumpur, Malaysia

## Abstract

Allele Specific Amplification with four primers (External Antisense Primer, External Sense Primer, Internal Nonfragrant Sense Primer, and Internal Fragrant Antisense Primer) and sensory evaluation with leaves and grains were executed to identify aromatic rice genotypes and their F_1_ individuals derived from different crosses of 2 Malaysian varieties with 4 popular land races and 3 advance lines. Homozygous aromatic (*fgr/fgr*) F_1_ individuals demonstrated better aroma scores compared to both heterozygous nonaromatic (*FGR/fgr*) and homozygous nonaromatic (*FGR/FGR*) individuals, while, some F_1_ individuals expressed aroma in both leaf and grain aromatic tests without possessing the *fgr* allele. Genotypic analysis of F_1_ individuals for the *fgr* gene represented homozygous aromatic, heterozygous nonaromatic and homozygous nonaromatic genotypes in the ratio 20 : 19 : 3. Genotypic and phenotypic analysis revealed that aroma in F_1_ individuals was successfully inherited from the parents, but either molecular analysis or sensory evaluation alone could not determine aromatic condition completely. The integration of molecular analysis with sensory methods was observed as rapid and reliable for the screening of aromatic genotypes because molecular analysis could distinguish aromatic homozygous, nonaromatic homozygous and nonaromatic heterozygous individuals, whilst the sensory method facilitated the evaluation of aroma emitted from leaf and grain during flowering to maturity stages.

## 1. Introduction

Aroma is the most important quality trait of aromatic rice which commands a higher price than nonaromatic rice. Thus, aromatic or scented rice plays a vital role in global rice trading [1–3]. Several chemical constituents including different volatile compounds are the major causes of aroma in cooked rice [4–6]. Moreover, Bradbury et al. [7] reported that a recessive gene (*fgr*) on chromosome 8 of rice which contains an 8 bp deletions and 3 Single Nucleotide Polymorphism (SNPs) produced a nonfunctional Betaine Aldehyde Dehydrogenase 2 (BADH2) enzyme resulting in aroma in rice. Many molecular markers such as RFLPs, RAPDs, STSs, and iso-enzymes have been developed for fragrant rice selection and identification [8]. Meanwhile, two types of molecular marker that is, Simple Sequence Repeat (SSR) and Single Nucleotide Polymorphism (SNP) were identified as promising marker, because they are genetically linked to aroma [6, 9–11]. In Addition, a perfect marker technique named Allele Specific Amplification (ASA) was developed by Bradbury et al. [12] for aroma genotyping and discriminating aromatic and nonaromatic rice. This technique was considered useful for selection of aromatic and nonaromatic rice genotypes in rice breeding programs [1]. In Malaysia, some constraints including high temperature during grain filling and ripening stage are slowing down the effectiveness of Maker-assisted selection for the improvement of aromatic rice varieties. So, aroma analysis throughout the life cycle using a combined sensory and molecular marker approach, may overcome these constraints by facilitating selection of the most appropriate parental materials for breeding programmes [13, 14]. In this study, we evaluated the efficacy of molecular markers and integration of sensory methods with these molecular markers for the detection of aroma in different rice genotypes.

## 2. Materials and Methods

### 2.1. Plant Materials

Six globally popular land races and eleven advance lines from International Rice Research Institute (IRRI) and three Malaysian cultivars from the Malaysian Agricultural Research Development Institute (MARDI) were used in this investigation (Table 1).

### 2.2. Crossing and Development of F_1_ Seeds

Crosses were made at the experimental field of the Genetic and Molecular Biology Division of the Institute of Biological Science, University of Malaya (71.43°E, 30.2°N & 122 meter above the sea level) from 15th June 2010 to 30th July 2010. Among twenty genotypes, two local genotypes MR 219 (Homozygous nonfragrant) and MRQ 50 (Homozygous fragrant) were used as the female and seven fragrant genotypes (Entry-7, Entry-11, Entry-13, Sadri, Gharib, Rato Basmati, and Ranbir Basmati) were used as male to produce F_1_ genotypes as they clearly demonstrated homozygous conditions by both ASA and sensory test.

### 2.3. Aroma Evaluation

The assessment of aroma in leaf was done according to Yeap et al. [15] which is a modified method of Sood and Siddiq [16] by using 0.2 g of leaf samples and cut into tiny pieces (<2 mm), but for grain aromatic test five (5) grains of each genotype were used following the method of Faruq et al. [14]. 

### 2.4. Extraction of DNA, PCR, and Genotyping

Young leaves were used for extracting total genomic DNA using Quick Extract plant DNA extraction solution from Epicentre biotechnologies (USA). Primers were designed as TTGTTTGGAGCTTGCTGATG (ESP), CATAGGAGCAGCTGAAATATATACC (IFAP), CTGGTAAAAAGATTATGGCTTCA (INSP), and AGTGCTTTACAAAGTCCCGC (EAP) based on Bradbury et al. [12] which were synthesised by Medigene (Malaysia). PCR was performed using 2.0 *μ*L of 10X reaction buffer (with 20 mM Mg^+^), 0.2 *μ*L of 10 mM dNTPs mix, 0.25 *μ*L of YEAtaq DNA Polymerase (Yeastem Biotech Co. Ltd., Taiwan), 5.0 *μ*L of DNA template, 0.4 *μ*L of each primer EAP, and ESP, 0.5 *μ*L of primer INSP and 0.5 *μ*L of IFAP, the total volume were 20 *μ*L. Amplification was carried out using a thermal cycler (C1000, BioRad, USA). Cycling conditions were performed 5 min at 94°C, followed by 35 cycles of 30 second at 94°C, 30 second at 53°C and 1 minutes at 72°C concluding with the final extension of 7 min at 72°C and hold at 4°C until recovery. Electrophoresis in 1.0% agarose gel and staining in ethidium bromide was done to analyse PCR products. PCR fragment size was estimated through 100 bp ladder (Vivantis, USA). The bands representing homozygous aromatic, homozygous nonaromatic, and heterozygous for *fgr* gene were analyzed by Allele Specific Amplification technique.

## 3. Results and Discussion

### 3.1. Allele Specific Amplification (ASA) of Parental Materials

**Figure 1:**
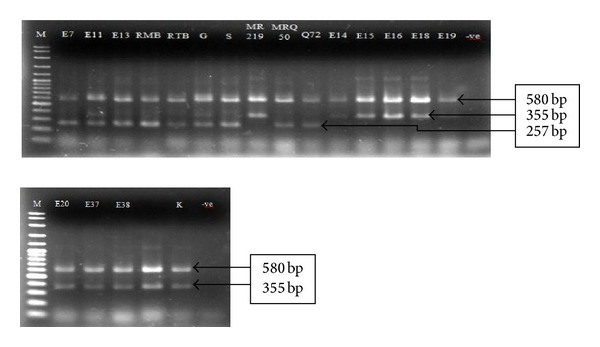
Aroma analysis for parental genotypes through single tube Allele Specific Amplification. E, Entry; RMB, Ranbir Basmati; RTB, Rato Basmati; G, Gharib; S, Sadri; K, Kasturi.

A set of 20 genotypes including 3 local checks (MRQ 50, MRQ 72, and MR 219) was chosen for aroma analysis of parental genotypes by Allele Specific Amplification. Among them Entry-11 and Gharib which scored 4 (Leaf & Grain aromatic test) for aroma were identified as homozygous for the fragrance gene (*fgr*) and genotype Sadri which also scored 4 (Leaf & Grain aromatic test) was identified as homozygous for the *fgr* gene. Moderate aroma (Mean aroma score 3) was found in Entry-7, Entry-13, Rambir Basmati, Rato Basmati, MRQ 50, and MRQ 72 which were also identified as homozygous for the *fgr* gene. On the other hand, Entry-14, Entry-15, Entry-16, Entry-18, Entry-19, Entry-20, Entry-37, Entry-38, and MR 219 which scored 1 (mean aroma) were identified as homozygous nonfragrant through ASA analysis. Surprisingly, Kasturi, with an aroma score 3 in the sensory test was scored as homozygous nonfragrant by ASA (Figure 1). Bounphanousay et al. [17] observed the same incident in a popular aromatic rice variety named Kai Noi Leuang from Laos.

ASA analysis of parent materials resulted in 580 bp sized bands representing the positive control, amplified by both EAP and ESP external primers, while 355 bp bands indicated a PCR product amplified from the nonfragrant allele by the External Antisense Primer (EAP) and Internal Nonfragrant Sence Primer (INSP). The 257 bp bands indicated a PCR product amplified from the fragrant allele (*fgr*) by the External Sence Primer (ESP) and Internal Fragrant Antisence Primer (IFAP). All genotypes produced a 580 bp band but only 9 genotypes (Entry-7, Entry-11, Entry-13, Rambir Basmati, Rato Basmati, Garib, Sadris, MRQ 50, and MRQ 72) showed bands of 257 bp indicating fragrant genotypes. The remaining genotypes produced 355 bp bands indicating nonfragrant genotypes. Previously, Bradbury et al. [12] mentioned that it is possible to differentiate nonfragrant from fragrant rice varieties and to identify fragrant homozygous, nonfragrant homozygous and nonfragrant heterozygous genotypes by using this method. 

### 3.2. Allele Specific Amplification (ASA) of F_1_ Hybrids

Among the twenty parental genotypes, seven aromatic genotypes (Entry-7, Entry-11, Entry-13, Rambir Basmati, Rato Basmati, and Garib and Sadris) were crossed with nonaromatic (MR 219) and aromatic (MRQ 50) local cultivars. The F_1_s derived from aromatic (7 genotypes) with nonaromatic (MR 219) crosses were slightly aromatic (Mean aroma score 2) and nonaromatic (Mean aroma score 1) represented heterozygous nonaromatic and homozygous nonaromatic individuals respectively. On the other hand, aromatic (7 genotypes) with aromatic (MRQ 50) crosses were produced homozygous aromatic (Mean aroma score 3) and slightly aromatic (Mean aroma score 2) F_1_ individuals. 

**Figure 2:**
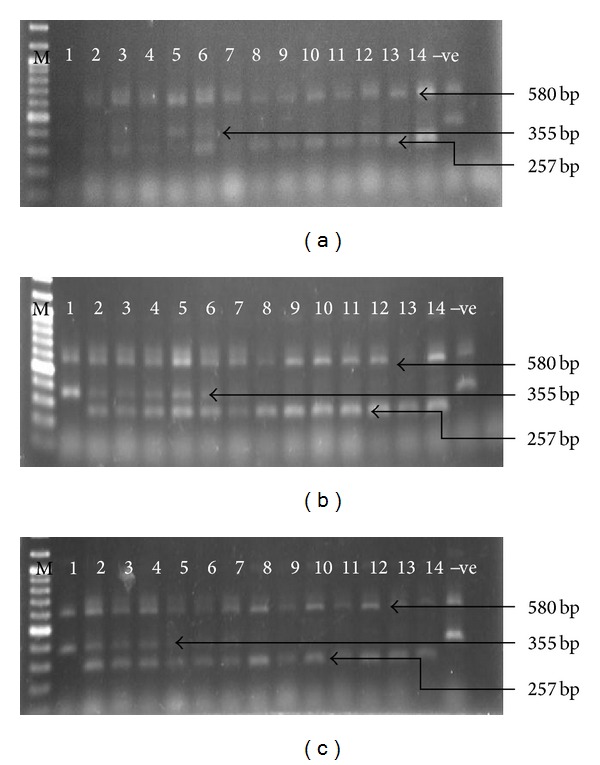
Fragrance analysis in F_1_ individuals using single tube Allele Specific Amplification (ASA). Lane 1–7, Gharib, Entry-7, Rambir Basmati, Entry-13, Rato Basmati, Entry-11, and Sadri crossed with MR 219, respectively; Lane 8–14, Rambir Basmati, Rato Basmati, Sadri, Gharib, Entry-11, Entry-13, and Entry-7 crossed with MRQ 50, respectively; (a), (b), and (c) representing 3 biological replicates (total of 42 seeds).

In Figure 2, Lanes 1–14 represented the F_1_ individuals derived from 14 different crosses with 3 replications, that is, 42 individuals. The 580 bp band was amplified from all individuals (positive control from ESP and EAP external primers). Bands of 355 bp (from amplification from the nonfragrant allele of *fgr* gene by primers EAP and INSP) and 257 bp (indicating presence of the *fgr *allele by the ESP and IFAP primers) were both amplified for 18 F_1_ individuals. The presence of only the 580 bp with the 335 bp band was observed for 3 F_1_ individuals whilst 21 individuals had only the 580 bp and 257 bp bands. The presence of 355 bp band indicated homozygous nonfragrant (without *fgr* allele) while 257 bp bands were represented the individuals as homozygous fragrant (homozygous for *fgr* gene). The individuals that represented both bands (355 bp and 257 bp) were identified as heterozygous nonfragrant individuals. Similar amplification pattern of fragrance (*fgr*) gene was observed by Bradbury et al. [12]. 

During this screening process, homozygous aromatic, heterozygous nonaromatic, and homozygous nonaromatic genotypes appeared in the ratio 20 : 19 : 3, which suggests that there are 20 aromatic and 22 (19 + 3) nonaromatic F_1_ individuals (Figure 2). Mohamad et al. [18] also observed a similar amplification pattern while used EAP, ESP, INSP, and IFAP primers (STS markers) in multiplex PCR condition and they identified 28 homozygous aromatic: 2 heterozygous nonaromatic: 45 homozygous nonaromatic, indicated 28 aromatic and 47 nonaromatic rice individuals. Meanwhile, another group of researchers, Bounphanousay et al. [17], detected 36 homozygous aromatic: 3 heterozygous nonaromatic: 17 homozygous nonaromatic whilst Sarhadi et al. [1] found 10 aromatic: 18 nonaromatic, also demonstrating the efficiency of these markers and 100% accuracy to detect this aroma allele. The results also confirmed the previous findings of Bradbury et al. [7] who demonstrated that the fragrance of basmati or jasmine rice were associated with the presence of a gene (*fgr*) on chromosome 8 of rice encoding nonfunctional BADH2. 

### 3.3. Aroma Evaluation through Sensory Methods

In this investigation, leaf and grain of selected parents (9 genotypes) and their F_1_ hybrid individuals were used for aroma evaluation (Table 2). From the leaf aromatic test, the highest mean aroma score was 3 while the lowest was 1. Individuals from five different crosses scored 3, from eight crosses scored 2 and from one cross scored 1. From grain aromatic test, the highest scoring was 2 and the lowest was 1. Individuals from 9 different crosses scored 2 and from 6 different crosses scored 1. Comparing the mean aroma scores from both methods, most produced a better leaf aroma score (Score 3) than the grain (score 2), except for the hybrids from MR 219/Rato basmati, where leaf aroma score was 1 and grain aroma score 2. F_1_s from MRQ 50/Gharib, MR 219/Entry-7, MR 219/Rambir Basmati, MR 219/Sadri had the same aroma score (score 2) in both leaf and grain. Genotypic analysis within F_1_ hybrids revealed that homozygous fragrant individuals produced better mean aroma score in leaf and grain than both heterozygous nonfragrant individuals and homozygous nonfragrant individuals, except for F_1_ from MRQ 50/Entry-11, MRQ 50/Sadri. Through comparison of both genotypic and phenotypic characteristics of aroma in F_1_ rice individuals, it was observed that aroma character from parents was successfully inherited to F_1_ individuals.

### 3.4. Integration of Sensory Methods and Molecular Markers for Detection of Aroma in Rice

Aromatic rice varieties emit aroma from their leaves, grains, and flowering organs at various stages of maturity [19]. There are many approaches used by researchers to determine the presence or absence of aroma in rice, such as evaluating aroma from leaves and grains with dilute KOH [16], analyzing the aroma using gas chromatography [20], and molecular markers related to rice aroma [12]. Sensory method facilitate the identification of aromatic and nonaromatic genotypes while molecular markers assist to identify specific allele but single tube allele specific amplification guides to identify zygosity (Homozygous or heterozygous for *fgr* gene) of individuals. In this study, we combined sensory tests and molecular marker methods for the detection of the presence or absence of aroma in parents and F_1_ hybrids. The F_1_ individuals, which were classified as having the aroma alleles (*fgr* gene) through molecular marker analysis, also showed presence of aroma in sensory tests. However, in less than 40% of the individuals which possessed aroma alleles and showed presence of aroma in sensory tests, the variation was from light aroma to strong aroma in leaf and grain aromatic tests. Less than 10% of the F_1_ individuals did not exhibit aroma in grain aromatic test while carrying *fgr* gene and producing leaf aroma. In another cases, around 30–40% of F_1_ individuals that were classified as homozygous nonfragrant (without *fgr *allele) but produced aroma in both the leaf and grain aromatic tests and <5% produced only grain aroma. Therefore, less than 50% of the F_1_ individuals that were classified as aromatic or nonaromatic rice by ASA were scored the same way in both leaf and grain using sensory detection. While more than 50% of the individual's demonstrated aroma by leaf or grain or ASA or in combination of the tests. These results indicated that only molecular marker analysis or sensory methods could not represent the complete aromatic conditions. Bounphanousay et al. [17] reported that the molecular marker results agreed well with chemical analysis in most of the rice varieties, except some contrasting results such as in a local aromatic rice variety, Kai Noi Leuang, which produced aroma but was identified as homozygous nonaromatic by molecular marker analysis. They suggested that different gene location might be responsible for the observed aroma or the presence of another major aromatic compound. Sarhadi et al. [1] reported coincidence between results from 1.7% KOH sensory testing and molecular marker analysis for the classification of aromatic and nonaromatic rice, but occasionally molecular markers could not classify heterozygous and homozygous genotypes. Yi et al. [2] also reported that variation in the sensory score may arise from minor genes or environmental factors and that some rice varieties may carry minor QTLs which have an influence on rice aroma. 

## 4. Conclusion

Aroma evaluation of rice genotypes is complicated in the tropical environment (countries like Malaysia) because of the large effects of environment and low sense of heritability. The integration of molecular markers and sensory tests can make the evaluation more effective. In allele specific amplification method, Entry-11, Gharib and Sadri was identified as homozygous for the fragrance allele (*fgr* gene), while the aroma scores were 4 in the sensory test. Genotypes Entry-13, Rato Basmati, Entry-7, Rambir Basmati, and two local checks MRQ 50 and MRQ 72 which scored 3, were also identified as homozygous for fragrance gene, but Kasturi with an aroma score of 3 in the sensory test was found as homozygous nonfragrant. However, homozygous aromatic F_1_ individuals possessed higher mean aroma score in leaf and grain compared to heterozygous and homozygous nonaromatic individuals. High aroma score was observed in F_1_s of MRQ 50/Entry-13, MRQ 50/Rambir Basmati and MRQ 50/Rato Basmati (leaf: 3; grain: 2). So, integration of sensory methods (Grain and Leaf aromatic test) along with allele specific amplification of 3 SNPs with 4 primers (ESP, EAP, INSP and IFAP) were observed as reliable, fast, and cost effective techniques in identifying parental materials and F_1_ individuals to evaluate rice aroma in this investigation.

## Figures and Tables

**Table 1:** Description of plant materials.

Source	Name	Type
International Rice Research Institute (IRRI**)**	Khau Dau Mali, Rato Basmati, Ranbir Basmati, Sadri, Gharib, Kasturi	Land races
	Entry-7 (IR 77734-93-2-3-2), Entry-11 (IR 78554-145-1-3-2) Entry-13 (IR 77512-2-1-2-2) Entry-14 (IR 77629-72-2-1-3) Entry-15 (M1-10-29 UL) Entry-16 (TOX 3226-5-2-2-2-2) Entry-18 (WAB 272-B-B-5-H5) Entry-19 (WAB 99-84) Entry-20 (WAB 337-B-B-15-H1) Entry-37 (PSB RC2 = IR 32809-26-3-3) Entry-38 (PSB RC18 = IR51672-62-2-1-1-2-3)	Advance lines
Malaysian Agricultural Research Development Institute (MARDI)	MRQ 72, MRQ 50 and MR 219	Malaysian cultivars

**Table 2:** Aroma performance of parents and F_1_ individuals in aromatic test (leaf and grain) and their genotypic expression.

Crosses	Leaf aroma			Grain aroma			Genotypic expression		
	♀ Parent	♂ Parent	F_1_	♀ Parent	♂ Parent	F_1_	♀ Parent	♂ Parent	F_1_
MR 219/Gharib	1	3	2	1	4	1	N	H	N
MR 219/RMB	1	3	2	1	3	2	N	F	H
MR 219/RTB	1	3	1	1	2	2	N	F	H
MR 219/Sadri	1	4	2	1	4	2	N	F	H
MR 219/Entry-7	1	3	2	1	3	2	N	F	H
MR 219/Entry-11	1	4	2	1	4	1	N	H	H
MR 219/Entry-13	1	3	2	1	2	1	N	F	H
MRQ 50/Gharib	3	4	2	2	4	2	F	H	F
MRQ 50/RMB	3	3	3	2	3	2	F	F	F
MRQ 50/RTB	3	3	3	2	2	2	F	F	F
MRQ 50/Sadri	3	4	2	2	4	1	F	F	N
MRQ 50/Entry-7	3	3	3	2	3	2	F	F	F
MRQ 50/Entry-11	3	4	3	2	4	1	F	H	H
MRQ 50/Entry-13	3	3	3	2	2	2	F	F	F

Genotypic expression F, N, and H represented homozygous fragrance, homozygous nonfragrance and heterozygous nonfragrance, respectively. The number 1 to 4 represented aroma condition such as 1: absence of aroma, 2: slight aroma, 3: moderate aroma, and 4: strong aroma and RMB for Ranbir Basmati, RTB for Rato Basmati.

## References

[1] Sarhadi WA, Hien NL, Zanjani M, Yosofzai W, Yoshihashi T, Hirata Y (2011). Comparative analyses for aroma and agronomic traits of native rice cultivars from Central Asia. *Journal of Crop Science and Biotechnology*.

[2] Yi M, Nwea KT, Vanavichit A, Chai-arree W, Toojinda T (2009). Marker assisted backcross breeding to improve cooking quality traits in Myanmar rice cultivar Manawthukha. *Field Crops Research*.

[3] Sakthivel K, Sundaram RM, Rani NS, Balachandran SM, Neeraja CN (2009). Genetic and molecular basis of fragrance in rice. *Biotechnology Advances*.

[4] Yajima I, Yanai T, Nakamura M, Sakakibara H, Hayashi K (1979). Volatile flavor components of cooked kaorimai (scented rice, *O. sativa japonica*). *Agricultural and Biological Chemistry*.

[5] Nijssen LM, Visscher CA, Maarse H, Willemsens LC, Boelens MH, Nijssen LM, Visscher CA, Maarse H, Willemsens LC, Boelens MH (1996). Volatile compounds in foods. *Qualitative and Quantitative Data*.

[6] Cordeiro GM, Christopher MJ, Henry RJ, Reinke RF (2002). Identification of microsatellite markers for fragrance in rice by analysis of the rice genome sequence. *Molecular Breeding*.

[7] Bradbury LM, Fitzgerald TL, Henry RJ, Jin Q, Waters DL (2005). The gene for fragrance in rice. *Plant Biotechnology Journal*.

[8] Lorieux M, Petrov M, Huang N, Guiderdoni E, Ghesquière A (1996). Aroma in rice: genetic analysis of a quantitative trait. *Theoretical and Applied Genetics*.

[9] Jin Q, Waters D, Cordeiro GM, Henry RJ, Reinke RF (2003). A single nucleotide polymorphism (SNP) marker linked to the fragrance gene in rice (*Oryza sativa* L.). *Plant Science*.

[10] Chen S, Wu J, Yang Y, Shi W, Xu M (2006). The *fgr* gene responsible for rice fragrance was restricted within 69 kb. *Plant Science*.

[11] Kibria K, Islam MM, Begum SN (2008). Screening of aromatic rice lines by phenotypic and molecular markers. *Bangladesh Journal of Botany*.

[12] Bradbury LM, Henry RJ, Jin Q, Reinke RF, Waters DL (2005). A perfect marker for fragrance genotyping in rice. *Molecular Breeding*.

[13] Golam F, NorZulaani K, Jennifer AH (2010). Evaluation of kernel elongation ratio and aroma association in global popular aromatic rice cultivars in tropical environment. *African Journal of Agricultural Research*.

[14] Golam F, Yin YH, Masitah A (2011). Analysis of aroma and yield components of aromatic rice in Malaysian tropical environment. *Australian Journal of Crop Science*.

[15] Yeap HY, Faruq G, Harikrisna JA Aroma analysis in few rice genotypes.

[16] Sood BC, Siddiq EA (1978). A rapid technique for scent determination in rice. *Indian Journal of Genetics and Plant Breeding*.

[17] Bounphanousay C, Jaisil P, Sanitchon J, Fitzgerald M, Hamilton NS (2008). Chemical and molecular characterization of fragrance in black glutinous rice from Lao PDR. *Asian Journal of Plant Sciences*.

[18] Mohamad O, Amiran N, Hadzim K, Hershey CH (2008). Molecular screening for aroma in rice. *Plant Breeding News*.

[19] Wongpornchai S, Sriseadka T, Choonvisase S (2003). Identification and quantitation of the rice aroma compound, 2-acetyl-1-pyrroline, in bread flowers (*Vallaris glabra* Ktze). *Journal of Agricultural and Food Chemistry*.

[20] Laksanalamai V, Ilangantileke S (1993). Comparison of aroma compound 2-acetyl-1-pyrroline in leaves from pandan. *Cereal Chemistry*.

